# Retraction: Novel fatty chain-modified GLP-1R G-protein biased agonist exerts prolonged anti-diabetic effects through targeting receptor binding sites

**DOI:** 10.1039/d3ra90124f

**Published:** 2024-01-04

**Authors:** Maorong Wang, Ping Yao, Minpeng Gao, Jian Jin, Yerong Yu

**Affiliations:** a Department of Endocrinology, West China Hospital, Sichuan University Chengdu Sichuan 610041 China 1100312222@vip.jiangnan.edu.cn yerongyu@scu.cn; b Department of Endocrinology, Affiliated Hospital of Hubei University for Nationalities Enshi 445000 Hubei China; c China Pharmaceutical University Nanjing Jiangsu P. R. China; d Jiangnan University Wuxi Jiangsu P. R. China

## Abstract

Retraction of ‘Novel fatty chain-modified GLP-1R G-protein biased agonist exerts prolonged anti-diabetic effects through targeting receptor binding sites’ by Maorong Wang *et al.*, *RSC Adv.*, 2020, **10**, 8044–8053, DOI: https://doi.org/10.1039/C9RA10593J.

The Royal Society of Chemistry hereby wholly retracts this *RSC Advances* article due to a number of concerns that were brought to our attention by a reader and undermine the integrity of the data.

There are many similarities with the content presented in ref. 1. But there are no overlapping authors. Ref. 1 was cited in this article as ref. 20, however, on multiple occasions in this article the authors have referred to ref. 20 as their own previous work.

In addition, scientific errors were raised by the reader and were verified by an independent expert. The method in the article is described as an autocrine-based screening method, however, it appears to be an affinity-based method. The claim that “six GLP-1R G-protein-biased peptides (named PX01–PX06)… were fused to the N-terminus of GLP-1(9-37) to generate six fusion peptides (PX07–PX012)” is incorrect. The claim that Exendin-4 is a native GLP-1R agonist is incorrect. The claim that the P5 peptide, first reported in ref. 1, has high immunogenicity as it shares 50% homology with GLP-1 is not supported.

The authors have not responded to the concerns and have not provided raw data. Due to the serious nature of these concerns, the editor has lost confidence in the integrity and reliability of this article.

The authors were informed about the retraction but did not respond.

Signed: Laura Fisher, Executive Editor, *RSC Advances*

Date: 13th December 2023

## Supplementary Material
